# Atypical Stroke-Like Presentation in a Critically-Ill Patient With Serratia marcescens Bullous Cellulitis

**DOI:** 10.7759/cureus.26404

**Published:** 2022-06-28

**Authors:** Sewar H Abuarqob, Brooke E Kania, Ariana R Tagliaferri, Sherif Roman, Rajapriya Manickam

**Affiliations:** 1 Internal Medicine, St. Joseph's University Medical Center, Paterson, USA; 2 Internal Medicine, St. Joseph’s University Medical Center, Paterson, USA; 3 Pulmonary and Critical Care Medicine, St. Joseph's University Medical Center, Paterson, USA

**Keywords:** sepsis, skin and soft tissue infections, gram-negative bacteria, serratia marcescens, bullous cellulitis

## Abstract

Skin and soft-tissue infections are common in critically ill patients, especially with gram-positive bacteria such as *Streptococcus* or *Staphylococcus* species. However, it is imperative to consider gram-negative infections in atypical presentations of bullous cellulitis, where patients do not initially respond to common empiric therapy for skin infections. *Serratia marcescens* is a gram-negative organism that manifests in nosocomial settings due to its affinity for moisture-rich environments. This bacteria is often difficult to treat due to extensive antibiotic resistance, and thus treatment is generally catered towards culture sensitivity. Rarely, this bacteria is an infective agent of infective endocarditis. We present a case of a 44-year-old gentleman who presented with stroke-like symptoms and was found to have bullous cellulitis with deep wound cultures growing *S. marcescens*. This case report highlights an atypical, severe presentation, and aims to provide a literature review of this rare manifestation of *S. marcescens* in skin and soft-tissue infections. We intend to improve rapid diagnosis and proper treatment for future critically-ill patients with skin and soft-tissue infections.

## Introduction

There is a low incidence of documented cases of patients with skin and soft-tissue infections primarily due to* Serratia marcescens* [1]. It is classified under the Enterobacteriaceae family and is difficult to treat due to its nature to thrive in moist environments, which subsequently leads to its antibiotic resistance [2]. The most common colonization site of the skin is on the lower extremities [2]. Immunocompromised patients or patients with diabetes mellitus, steroid use, indwelling catheters, and/or intubated patients are at the highest risk of being afflicted [3,4]. Depending on the patient's comorbidities, the prognosis can be poor with a 50% mortality rate if necrotizing fasciitis develops [2]. Herein, we present a case of a young male, who was critically ill, initially presented with stroke-like symptoms, and deteriorated quickly from severe bullous cellulitis due to *S. marcescens*.

## Case presentation

**Figure 1:**
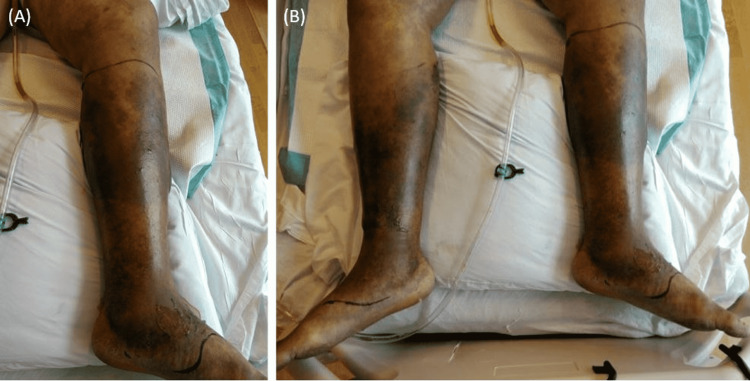
Photographs of the patient’s bilateral lower extremities demonstrating skin changes due to cellulitis. Significant hyperpigmentation and venous stasis changes of the bilateral lower extremities from ankles to knees with pitting edema bilaterally, and without evidence of crepitus were noted.

The patient was a 44-year-old male with a past medical history of non-insulin-dependent diabetes mellitus, intravenous heroin use, and hepatitis C who presented to the emergency department (ED) with complaints of slurred speech, bilateral lower leg swelling, and pain that started earlier that day. Initial vitals showed hypotension with a blood pressure of 109/68 mmHg, tachycardia at 160 beats per minute, tachypnea with a respiratory rate of 40 breaths per minute, and hypothermia with a temperature of 34.9 degrees Celsius. On arrival, a stroke code was activated. However, The National Institutes of Health stroke scale (NIHSS) was 5 with a Glasgow coma score of 15. On physical exam, the patient was alert and oriented to time and place. His speech was moderately slurred and he had bilateral lower extremities weakness. Dorsalis pedis pulses (appreciated via Doppler ultrasound) were weak, also noted were venous stasis changes, pitting edema, and no evidence of crepitus (Figure 1). 

**Figure 2:**
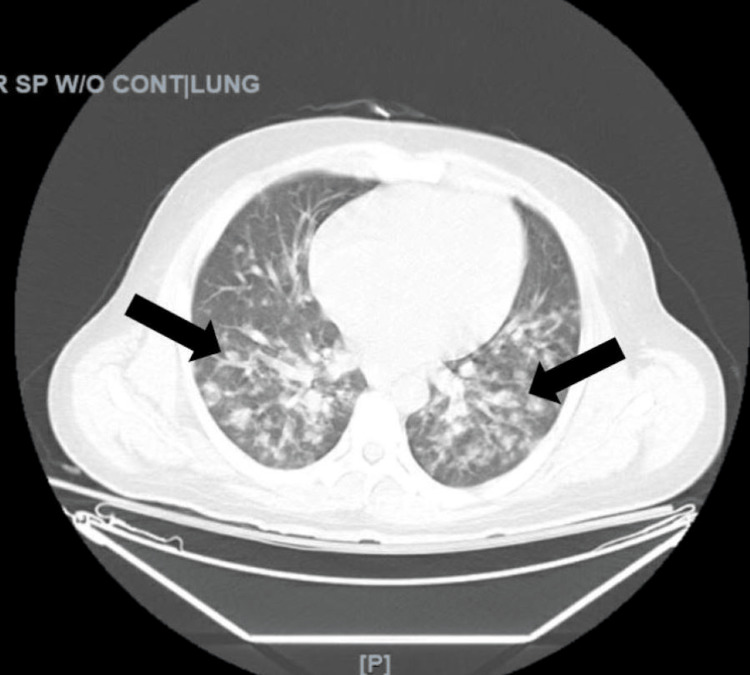
CT of the chest showing nodular infiltrates in both lung fields, suggestive of septic emboli.

**Figure 3:**
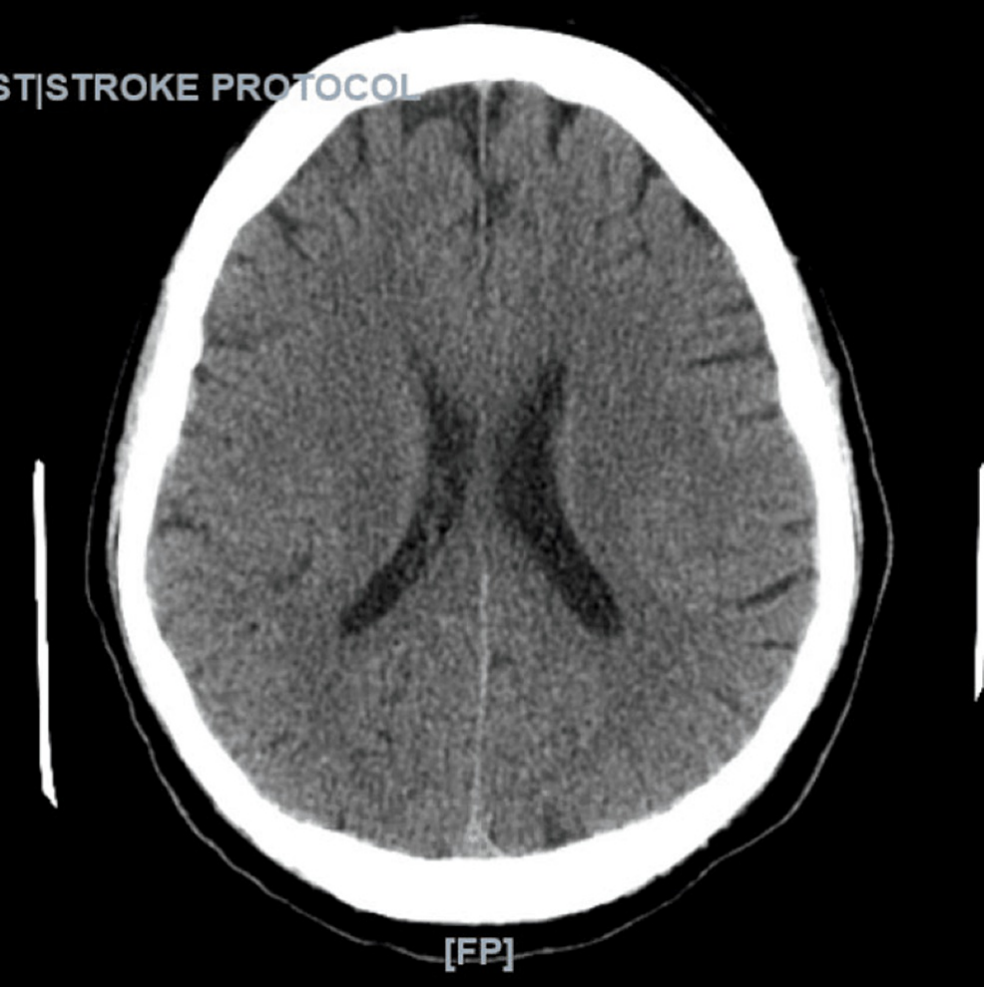
CT of the head without contrast (axial view) was negative for intracranial hemorrhage. There was no evidence of an acute cortical infarct, mass effect, midline shift or, hydrocephalus.

Laboratory studies were significant for severe metabolic acidosis (pH 7.16 on venous blood gas), leukocytosis with a white blood cell count of 12.6x10^3/mm3, thrombocytopenia with a platelet count of 24K/mm3, acute kidney injury with a creatinine 2.65 mg/dL, conjugated hyperbilirubinemia (total bilirubin 17.1 mg/dL, direct bilirubin 13.7 mg/dL), and lactic acidosis of 18.1 mmol/L. Computerized Tomography (CT) of the chest, abdomen, and pelvis was significant for possible septic emboli in the lungs (Figure 2). All other work-up including urinalysis, chest radiograph, and CT of the brain, was unremarkable (Figure 3). 

 

Due to the limited ability to protect his airways and severe metabolic derangements, the patient was subsequently intubated and admitted to the medical intensive care unit for further evaluation and management of septic shock.

He was initially treated empirically with vancomycin and piperacillin-tazobactam, which were later escalated to vancomycin and meropenem due to culture positivity for gram-negative rods. Surgery was consulted out of concern for necrotizing fasciitis. Final blood cultures, deep wound cultures, and urine cultures were positive for *S. marcescens*.

Bedside point of care ultrasound was remarkable for adequate left ventricular contractility, possible mitral valve thickening, and poor visualization of the tricuspid valve. Transesophageal echocardiogram (TEE) was pending due to suspected infective endocarditis; however, the patient was in severe septic shock, with worsening lactic acidosis, and despite maximum efforts, went into asystole, and eventually expired within 24 hours of his presentation.

## Discussion

The incidence of cutaneous infections secondary to *S. marcescens* is reportedly very low [1]. A retrospective analysis of 15 patients with *S. marcescens* soft-tissue infections noted the median age of patients to be 60 years old, with a range from eight years to 88 years of age [2]. Of these patients, 62% were male, and the most common site of* S. marcescens* infections was in the lower extremities, seen in 75% of the cases [2]. It has been shown that patients who are immunosuppressed, chronically utilize steroids, have indwelling catheters, are dependent on mechanical ventilation, have a history of trauma, renal failure, diabetes mellitus, chronic leg ulcers, or recently received broad-spectrum antibiotics may be predisposed to infections with this bacteria [3,4]. Our patient represents the majority of patients as a male, with a slightly younger presentation compared to the median age identified in the study above. His past medical history of untreated hepatitis C, diabetes mellitus, and intravenous drug use placed him at a higher risk for developing this lethal infection.

*Serratia marcescens*, part of the Enterobacteriaceae family, encompasses a gram-negative bacillus, a facultative anaerobe, that can survive in nutrient-poor environments such as disinfectant agents, water, or pipes [5,6]. Due to contamination of *S. marcescens* in common hospital environments, appropriate sterile precautions should be taken to prevent nosocomial infections [6]. These bacteria commonly colonize within the respiratory, gastrointestinal, or genitourinary tracts and can lead to endocarditis or sepsis [5]. Patients who develop bullous cellulitis most commonly have positive culture results for *Streptococcus pyogenes*, however,* Staphylococcus aureus* is another etiology [6]. Current literature does not suggest that *S. marcescens* is a common etiology of soft-tissue or skin infections [7]. Our patient presented with bullous cellulitis before intubation and thus a nosocomial infection is unlikely. As he had numerous cultures positive for *S. marcescens*, it is difficult to determine the origin of his sepsis. 

Unfortunately, *S. marcescens* confers high antibiotic resistance due to its chromosomal beta-lactamase [8]. Additionally, due to protein variations of the bacterial outer membrane which works to inhibit drug permeability, this bacteria also confers resistance to fluoroquinolones [9]. Instead of this, treatment of *S. marcescens* infection based on the antibiogram data and current data elucidates that carbapenems, aztreonam, as well as third and fourth-generation cephalosporins are the agents of choice for* S. marcescens *infections [2]. Due to the severity of our patient’s illness, empiric antibiotics were administered whilst awaiting culture sensitivity. In a retrospective review, approximately 50% of patients who were initiated on any of the aforementioned antibiotics were not sensitive to* S. marcescens*, further indicating the importance of culture sensitivity data when directing management [2]. This is particularly difficult in soft-tissue and skin infections, as they are often caused by gram-positive bacteria and thus treatment is typically tailored towards those causative organisms which are subsequently ineffective in treating *S. marcescens* [10]. 

In the review mentioned above, the overall mortality rate of patients with *S. marcescens* soft-tissue and skin infections is approximately 31%, which decreases to 19% after excluding confounding causes of mortality. With concomitant complications of *S. marcescens* infections such as necrotizing fasciitis, the mortality rate increases to 50% [2]. Although this was a concern in our patient, he ultimately deteriorated before a complete evaluation of necrotizing fasciitis. Our patient’s demise may also have been from pulmonary septic emboli that may have spread to the brain leading to his neurological deficits, or from *S. marcescens* endocarditis. Unfortunately, this information will never be conclusive due to the patient’s rapid deterioration. 

## Conclusions

Although rarely documented, *S. marcescens* should be included in the differential when investigating skin and soft-tissue infections with gram-negative bacterial etiology, especially in immunocompromised patients or those with concomitant comorbidities listed above. One should consider the difficulty in treating this organism and remember that empiric therapy with third or fourth-generation cephalosporins is recommended until culture sensitivity results. Additional research is warranted to determine faster and more accurate means of diagnosis to avoid rapid deterioration and to prevent adverse patient outcomes.
